# TLR7/8 signalling affects X-sperm motility via the GSK3 α/β-hexokinase pathway for the efficient production of sexed dairy goat embryos

**DOI:** 10.1186/s40104-021-00613-y

**Published:** 2021-08-03

**Authors:** Fa Ren, Huaming Xi, Yijie Ren, Yu Li, Fei Wen, Ming Xian, Mengjie Zhao, Dawei Zhu, Liqiang Wang, Anmin Lei, Jianhong Hu

**Affiliations:** 1 Key Laboratory of Animal Genetics, Breeding and Reproduction of Shaanxi Province, College of Animal Science and Technology, Northwest A&F University, No. 22 Xinong Road, Yangling, Shaanxi 712100 People’s Republic of China; 2 College of Veterinary Medicine, Northwest A&F University, Yangling, 712100 Shaanxi China

**Keywords:** Dairy goat, Glycogen synthase kinase α/β (GSK3 α/β), Sexing control, Sperm, Toll-like receptor 7/8 (TLR7/8)

## Abstract

**Background:**

Goat milk is very similar to human milk in terms of its abundant nutrients and ease of digestion. To derive greater economic benefit, farmers require more female offspring (does); however, the buck-to-doe offspring sex ratio is approximately 50%. At present, artificial insemination after the separation of X/Y sperm using flow cytometry is the primary means of controlling the sex of livestock offspring. However, flow cytometry has not been successfully utilised for the separation of X/Y sperm aimed at sexing control in dairy goats.

**Results:**

In this study, a novel, simple goat sperm sexing technology that activates the toll-like receptor 7/8 (TLR7/8), thereby inhibiting X-sperm motility, was investigated. Our results showed that the TLR7/8 coding goat X-chromosome was expressed in approximately 50% of round spermatids in the testis and sperm, as measured from cross-sections of the epididymis and ejaculate, respectively. Importantly, TLR7/8 was located at the tail of the X-sperm. Upon TLR7/8 activation, phosphorylated forms of glycogen synthase kinase α/β (GSK3 α/β) and nuclear factor kappa-B (NF-κB) were detected in the X-sperm, causing reduced mitochondrial activity, ATP levels, and sperm motility. High-motility Y-sperm segregated to the upper layer and the low-motility X-sperm, to the lower layer. Following in vitro fertilisation using the TLR7/8-activated sperm from the lower layer, 80.52 ± 6.75% of the embryos were XX females. The TLR7/8-activated sperm were subsequently used for in vivo embryo production via the superovulatory response; nine embryos were collected from the uterus of two does that conceived. Eight of these were XX embryos, and one was an XY embryo.

**Conclusions:**

Our study reveals a novel TLR7/8 signalling mechanism that affects X-sperm motility via the GSK3 α/β-hexokinase pathway; this technique could be used to facilitate the efficient production of sexed dairy goat embryos.

**Supplementary Information:**

The online version contains supplementary material available at 10.1186/s40104-021-00613-y.

## Background

Sexing control is an important technique in livestock breeding that facilitates the production of offspring of a specific sex, according to the farmers’ preference, through specific interventions [1]. In livestock production, the demand for specific animal products often results in the need for biased sex control of animal offspring [2]. For example, the production of goat milk requires female dairy goats. Artificial insemination (AI) after the separation of X and Y chromosome-bearing sperm is one of the primary means of sex control of livestock offspring [3]. In fact, scientists have long been interested in separating X- and Y-sperm [4, 5]; however, flow cytometry, which uses differences in sperm DNA to separate X- and Y-sperm, is thus far the only recognised method of sex control in livestock [6], and this method has only been successfully applied to cattle [7]. Essentially, the chromosomal DNA content of X-sperm is 4.4 ± 0.03% higher than that of Y-sperm in goats; thus, in principle, goat sperm are more amenable to be separated via flow cytometry than cattle sperm [8, 9]. Even so, the use of flow cytometry to separate X- and Y-sperm in goats has resulted in low sperm motility and poor pregnancy rates [10]. This outcome might be due to species-specific differences in mammalian sperm. Considering this, we aimed to find a new method to separate dairy goat X- and Y-sperm without flow cytometry.

Mammalian spermatogenesis is a highly complex and orderly process that ultimately produces X-chromosome-bearing sperm (X-sperm) and Y-chromosome-bearing sperm (Y-sperm), which determine the offspring sex [6]. X- and Y-sperm are equally amenable to in vivo and in vitro fertilisation (IVF) procedures. Studies have shown several differences between X- and Y-sperm, including size and density, DNA content, and motility under various environmental conditions [11, 12]. At present, a few convincing reports regarding the separation of X- and Y-sperm based on the basic and physiological differences between the two have been published. Recently, Umehara et al. reported that toll-like receptors 7 and 8 (TLR7 and TLR8), which are membrane-associated receptor proteins, were specifically expressed at the X-sperm tail and not in the Y-sperm. Interestingly, ligand activation of TLR7/8 selectively suppressed the mobility of the X-sperm, but not that of the Y-sperm, thereby allowing for the separation of X- and Y-sperm in mice [13]. The work of Umehara et al. opens up exciting avenues for the further control of offspring sex in mammals on the basis of differences in the sperm surface-specific proteins [14]. In particular, it provides a new reference for the sex control of livestock offspring. However, the effects of TLR7/8 signalling in X- and Y-sperm have not been elucidated in dairy goats and other livestock.

Goat milk is nutritionally similar to human milk due to its abundance of nutrients and ease of digestion [15]. The current population of dairy does showing high milk production yields is insufficient to meet the growing human demand for goat milk. In addition, the value of buck kids is significantly lower than that of doe kids in dairy goat breeding. The ratio of bucks to does produced by natural mating and reproduction is approximately 1:1, and the breeding of surplus male dairy goats increases the production costs [16]. Thus, dairy goat sperm sexing technologies could be used to produce a higher ratio of doe kids to reduce breeding costs. However, there have been no reports detailing the practical and successful separation of X- and Y-sperm in dairy goats. In this study, the localisation of TLR7/8 in the testes, epididymis, and sperm of dairy goats was evaluated. The effects of the incubation of sperm with a TLR7/8 ligand (R848) on sperm motility parameters, mitochondrial activity, and ATP levels were evaluated. We sought to prove that TLR7/8 was exclusively expressed in goat X-sperm. The mechanism whereby TLR7/8 affects X-sperm motility was also investigated. Based on this finding, we developed a novel yet simple goat-sperm sexing technology that could be used to selectively separate X- and Y-sperm, and to efficiently produce sex-selected embryos in dairy goats.

## Methods

### Ethics statement

All experiments in the current study were approved by the Institutional Animal Care and Use Committee of the Northwest A&F University, Shaanxi, China.

### Chemicals and reagents

Unless otherwise stated, all chemicals and reagents used in the present study were purchased from Sigma-Aldrich, China. The R848 (TLR7/8 ligand) was obtained from Novus Biologicals (Littleton, CO, USA).

### Semen collection and swim-up test

**Fig. 1:**
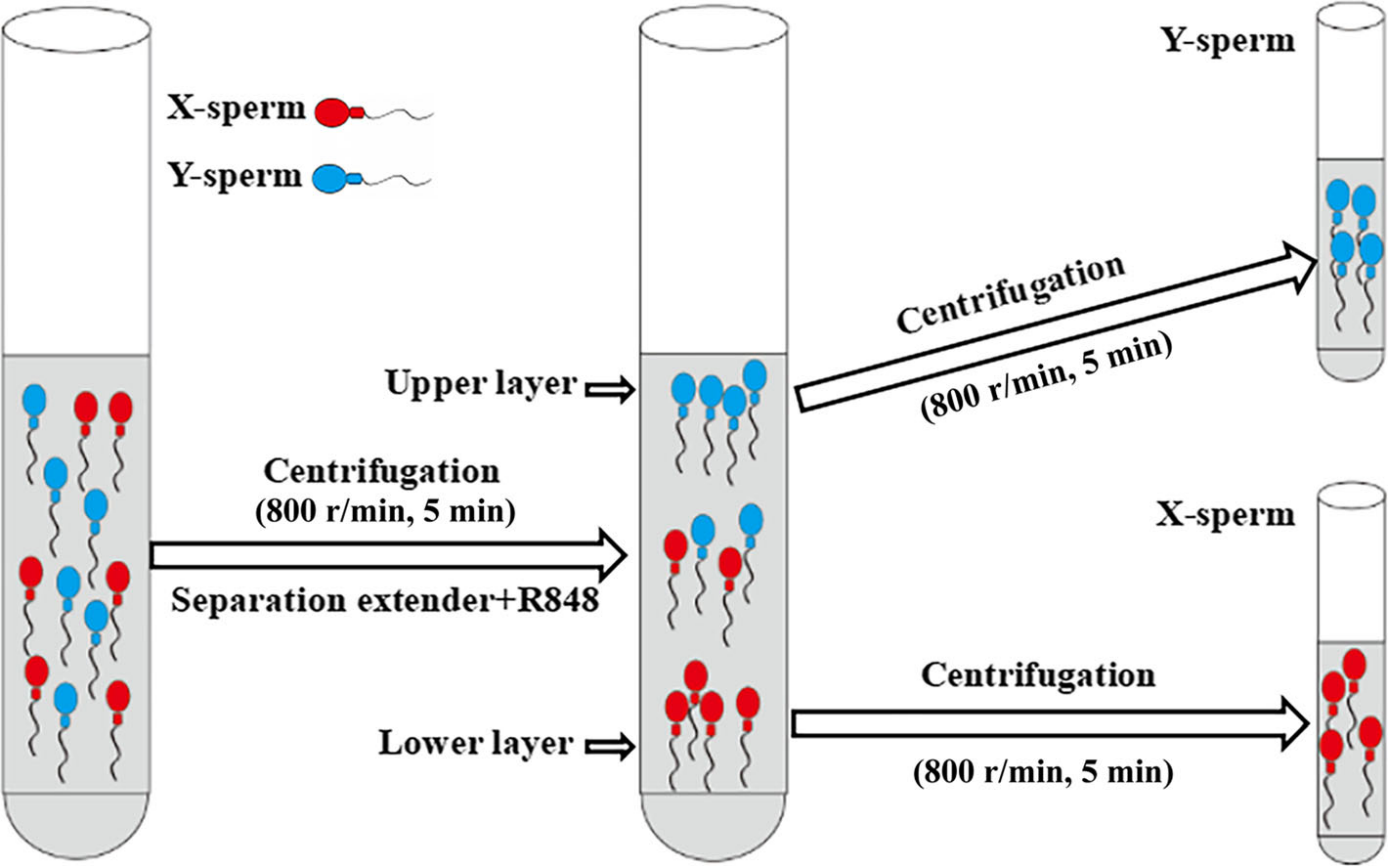
The technical diagram of goat X/Y sperm separation method using R848. The goat semen were centrifuged at 800 r/min for 5 min, resuspended in seperation extender and R848, and incubated on 37 °C. Next, sperm in the upper (Y-sperm) and lower layers (X-sperm) was evaluated

Semen was collected twice weekly from six Guanzhong dairy goat bucks (2–3 years old) using an artificial vagina at the Shaanxi Aonike Guanzhong Dairy Goat Breeding Farm, Fuping, Shaanxi, China. The ejaculate was transported to the lab, and sperm quality was assessed within 30 min. All semen samples with sperm motility > 75% were mixed to avoid the influence of the individual bucks used in the study. The swim-up test is shown in Fig. 1; it was performed as previously described [13]. The sperm were incubated in a goat semen extender [17] (3 mL per group; 1 × 10^8^ sperm/mL), with several concentrations of R848 for different time intervals at 37 °C. Next, the percentages of sperm in the upper (1 mL) and lower (1 mL) layers were evaluated (number of sperm in upper or lower layer/total sperm). The separated sperm were used for further analysis.

### Sperm motility parameter assessment using the computer-aided sperm analysis (CASA) system

Sperm were incubated in a goat semen extender containing 1 μmol/L R848 at 37 °C for 30 min. Then, aliquots (50 μL) of each treated semen sample were diluted with 50 μL of base extender in PCR tubes. After the mixed samples (100 μL) were incubated in a water bath (37 °C) for 5 min, each semen sample (10 μL) was transferred to a pre-warmed glass slide (37 °C) containing a coverslip (CELL-VU® DRM-600, Millennium Sciences lnc. New York, USA). Sperm motility parameters were evaluated using a computer-aided sperm analysis (CASA) system (HVIEW-SSAV8.0, FuZhouHongShiYe Software Technology Co., Ltd., China) [18]. The standard parameters were set at 30 frames/s and sperm tracks were captured at 60 Hz. The measured sperm motility parameters included sperm motility, average path velocity (VAP), average straight-line velocity (VSL), and average curvilinear velocity (VCL). We defined sperm motility as the percentage of sperm with straightness of path (STR) ≥ 75% and VSL > 25 μm/s. A minimum of 500 sperm were observed from at least five randomly selected fields using a phase-contrast microscope (Nikon 80i; Nikon, Tokyo, Japan). All experiments were replicated five times, and performed between July and November 2020.

### Immunofluorescence (IF) of testis and epididymis

Three Guanzhong dairy goat bucks (8 months old) were euthanised after overnight fasting with neck vein injections of pentobarbital (50 mg/kg body weight; Release; WDT, Garbsen, Germany) and xylazine (0.5 mg/kg body weight; Xylosol; Ogris Pharme, Wels, Austria) [19]. Then, their testes and epididymis were collected and fixed in 4% (w/v) paraformaldehyde overnight and embedded in paraffin, as described previously [13, 20]; next, the tissue sections of the testis and epididymis were probed with an anti-TLR7 antibody (bs-6601; Bioss Antibodies, Woburn, MA, USA) and anti-TLR8 antibody (ab180610; Abcam, Cambridge, MA, USA) diluted at a ratio of 1:200. After washing with PBS, the antigens (TLR7 and TLR8) were visualised using Cy3-conjugated goat anti-rabbit IgG (ab6939; Abcam), 30 μL fluorescein isothiocyanate-peanut agglutinin (FITC-PNA, 100 μg/mL in PBS), a marker of acrosome in round spermatid and sperm [21], and 1 μg/mL DAPI, which was used to stain the nuclei. Stained sections were monitored and photographed using an epifluorescence microscope (Nikon Eclipse C1; Nikon, Tokyo, Japan). For the testes, three cross-sections of seminiferous tubules with more than 100 round spermatids in each tubule were randomly selected using a phase-contrast microscope (Nikon Eclipse C1). Round spermatids of goat testes were stained with TLR7/8 (red) and PNA (green), and the proportion of TLR7/8-positive cells (%) was calculated as follows: (the number of PAN+ and TLR7+ cells/number of PAN+ positive cells) ×100. Consistently, three cross-sections of epididymis tissues with more than 300 sperm in each lumen were randomly selected using a phase-contrast microscope (Nikon Eclipse C1). Sperm of the epididymis tissues were stained with anti-TLR7/8 (red) and PNA (green), and the proportion of TLR7/8-positive sperm (%) was calculated as follows: (the amount of PAN+ and TLR7+ sperm/PAN+ sperm) ×100. Visualisations were performed using a DAPI ultraviolet excitation wavelength of 330–380 nm, with an emission wavelength of 420 nm (blue light emission); an FITC excitation wavelength of 465–495 nm with an emission wavelength of 515–555 nm (green light emission); and a Cy3 excitation wavelength of 510–560 with an emission wavelength of 590 nm (red light emission). All experiments were completed in triplicate from August–December 2019.

### Immunofluorescence of sperm

The sperm samples obtained from the dairy goat bucks was spread over slides, air-dried, and fixed in 100% methanol for 10 min. After air-drying, the sample slides were rinsed with PBS three times for 5 min (each time). The sperm samples were then permeabilised with 0.5% (v/v) Triton X-100/PBS at room temperature for 30 min. The sample slide was rinsed with PBS three times for 5 min (each time). The sperm were incubated with a blocking solution (5% BSA) at 37 °C for 30 min. Then, they were probed with anti-TLR7 or anti-TLR8 antibodies at a ratio of 1:100 and incubated overnight at 4 °C. After washing with PBS, the antigens were visualised with Cy3-conjugated goat anti-rabbit IgG (1:2,000; ab6939, Abcam) and 1 μg/mL DAPI. Sperm staining was monitored and photographed using an epifluorescence microscope (Nikon Eclipse C1). The experiments were completed in triplicate from September–December 2019 and July–November 2020.

### Analysis of sperm membrane integrity

The membrane integrity of the upper- and lower-layer sperm was evaluated after incubation in an extender containing 1 μmol/L R848 at 37 °C for 30 min. Sperm membrane integrity was evaluated using SYBR-14/propidium iodide (PI), according to a previously described procedure [22]. Briefly, the sperm samples (100 μL) were stained using 0.1 μL of SYBR-14 working solution (100 μmol/L in DMSO) for 10 min at 37 °C, and then using 0.5 μL of propidium iodide (PI) working solution (2.4 mol/L in water) for 10 min at 37 °C. Sperm staining was monitored and photographed using an epifluorescence microscope (Nikon Eclipse C1). More than 200 spermatozoa were assessed per slide. Five replicates of each semen sample were evaluated by the same observer. All experiments were completed in September 2020.

### Analysis of sperm acrosome integrity

The acrosome integrity of the upper- and lower-layer sperm was evaluated after incubation in an extender containing 1 μmol/L R848 at 37 °C for 30 min. In accordance with the method described by Ren et al. (2019) [17], acrosome integrity was measured using fluorescein isothiocyanate-peanut agglutinin (PNA-FITC) staining. Briefly, sperm samples (30 μL) were spread over slides, air-dried, and fixed in 100% methanol for 10 min. The fixed sperm were then stained using 30 μL of PNA-FITC (100 μg/mL in PBS), incubated at 37 °C for 30 min, and rinsed thrice with PBS. Nuclear staining was performed using 1 μmol/L DAPI. The surface dye was washed three times using PBS and air-dried in the dark. The spermatozoa staining was monitored, and the stained spermatozoa were photographed using an epifluorescence microscope (Nikon Eclipse C1). More than 100 spermatozoa were assessed per field, and five randomly selected fields were assessed for each sample. Five replicates of each semen sample were evaluated by the same observer. The experiments were completed in September 2020.

### Analysis of sperm mitochondrial activity and ATP levels

The mitochondrial activity changes in the sperm were evaluated using a JC-1 lipophilic cation (5,5′,6,6′-tetrachloro-1,1′,3,3′-tetraethylbenzimidazolcarbocyanine iodide) Mitochondrial Membrane Potential Detection Kit (Beyotime Institute of Biotechnology, Haimen, China), according to the manufacturer’s instructions, as described by Li et al. (2020) [23]. Briefly, sperm samples (2 × 10^6^/mL) were stained with 28 μL of JC-1 (stock solution) in PBS (final volume, 100 μL). After incubation at 37 °C for 30 min in the dark, the samples were centrifuged at 600 *g* for 5 min at 4 °C and washed with JC-1 buffer twice. After resuspension in JC-1 buffer, the sperm samples were immediately analysed using a flow cytometer (FACSCalibur; BD Biosciences, San Jose, CA, USA), with excitation wavelengths of 490 nm and 525 nm and emission wavelengths of 530 and 590 nm for the JC-1 monomer and the JC-1 polymer, respectively. Sperm mitochondrial activity was calculated using the following equation:

Sperm mitochondrial activity (%) = the value of JC-1 polymer/(JC-1 monomer + JC-1 polymer) ×100%.

Three replicates were evaluated for each sperm sample.

ATP levels of dairy goat sperm were assessed using an ATP Assay Kit (Beyotime Institute of Biotechnology), according to the manufacturer’s instructions, and as described by Li et al. (2020) [23]. Briefly, 500 μL of semen (8 × 10^7^ sperm/mL) was centrifuged at 4 °C and resuspended in ATP assay lysate (200 μL) to release the intracellular ATP. The sperm sample was then centrifuged at 12,000 *g* at 4 °C for 5 min and the supernatant was aspirated. The ATP standard solution (0.5 mmol/L) was diluted to concentrations of 0.01, 0.03, 0.1, 0.3, 1, 3, and 10 μmol/L in succession using ATP assay lysate. The supernatants (50 μL) and standards (50 μL) were added to a 100-μL ATP detection working solution in opaque 96-well plates. The fluorescence intensity of the samples was detected using a multi-detection microplate reader (Bio Tek Synergy H1; Bio Tek, Winooski, VT, USA). Three replicates were evaluated for each sperm sample. All experiments were completed between July and November 2020.

### In vitro fertilisation (IVF) using the lower layer TLR7/8-activated sperm

Goat ovaries were obtained from a local abattoir and transported to the laboratory within 4 h in 0.9% sodium chloride solution at 38.5 °C. After removing the connective tissues, fat mass, and fallopian tubes, the ovaries were soaked in 75% alcohol for 30 s and washed 3 times with a 0.9% sodium chloride solution preheated to 38.5 °C. The ovaries were placed into a 60-mm petri dish containing TCM-199 medium [24] with 6 mg/mL BSA (washing medium) and fixed with tweezers. Subsequently, the follicles on the surface of the ovary were lacerated with a scalpel. The follicles were washed with the oocyte collection fluid to ensure the complete flow of the follicular fluid. The cumulus-oocyte complexes (COCs) were collected under a stereo microscope (LECIA M165 FC; Lecia; German) using a self-made mouth pipette, and COCs with three layers of cumulus cells were used in this experiment. Thirty to forty COCs were placed in a 200-μL drop of BO-IVM medium (IVF Bioscience, Falmouth, United Kingdom), and incubated for 24 h at 38.5 °C and 5% CO_2_. TLR7/8-activated sperm (50 μL; 2 × 10^7^ spermatozoa/mL) were added to the 30–40 COCs in a 500-μL six-well dish containing BO-IVF (IVF Bioscience) medium and mineral oil and incubated for 18 h at 38.5 °C and 5% CO_2_. Subsequently, the unfertilised sperm and cumulus cells were removed from the zygotes using 0.1% hyaluronidase PBS. Next, the zygotes were transferred to BO-IVC medium (IVF Bioscience) for culture. After 7 d, the blastocysts were identified and collected, and their sex was determined using PCR. The experiments were performed in triplicate and were completed between July and November 2020.

### Artificial insemination (AI) using the lower layer TLR7/8-activated sperm

A CIDR (CIDR®, EaziBreed; Zoetis, Parsippany-Troy Hills, NJ, USA) device was intravaginally placed in the donor Guanzhong dairy goat does (*n* = 6) on day 0. On day 14, the goats were injected with FSH (Folltropin-V®; Bioniche Animal Health, Belleville, Canada), starting in the morning. In total, 180 mg of FSH was injected over 3 d (50, 50, 30, 30, 10, and 10 mg) at 12-h intervals. The CIDR device was removed from the does on day 17. After 24 h, the donor goats were intra-cervically inseminated with the lower-layer sperm (incubated with 1 μmol/L R848 at 37 °C for 30 min), as described in our previous report [17]. Briefly, an opener was used to open the vagina and observe the changes to evaluate the oestrus period of the donor goat. The oestrus period was defined by the presence of more viscous secretions in the vagina and a congested, loose, and open cervix. Two inseminations after the oestrus period (12 h apart, 0.5 mL with 5 × 10^7^ spermatozoa each time) were performed and the resulting embryos were collected after 5 d for sex determination using PCR. Transcervical embryo recovery was performed as described by Santos et al. (2020) [25]. The experiments were completed in December 2020.

### Sex determination of goat embryos by PCR

Each of the blastocysts was transferred to PBS in 0.01% BSA for washing in vitro, and then individually placed in a 0.2-mL enzyme-free centrifuge tube and dispensed with 2.5 μL of genome extract (1 mg/mL Proteinase K, 0.5% Triton X-100, 50 mmol/L Tris-HCl, pH = 8.0). Single blastocysts were then placed in a Polymerase Chain Reaction (PCR) machine (T100; Bio-Rad; United States) and incubated at 65 °C for 3 h and at 95 °C for 10 min. PCR analyses of single blastocysts were performed with KOD FX Neo (KFX-201, Toyobo Co., Ltd., Life Science Department, Osaka, Japan), according to the manufacturer’s instructions. Primer sets recognising the Y- (*SRY*) and X-chromosomes (*B-ACTIN*) were generated and used for the PCRs (Table 1). The following PCR amplification conditions were used: initial denaturation at 95 °C for 5 min and 35 cycles of denaturation at 95 °C for 30 s, annealing at 55 °C for 30 s, and elongation at 68 °C for 40 s, with a final extension at 68 °C for 5 min. Subsequently, the PCR products were visualised by 2% agarose gel electrophoresis. Each lane represented an embryo, except for the Ma and Fe lanes, which represented the DNA of control male (Ma) and female (Fe) dairy goats, respectively. Female embryos were identified by the production of one band (*B-ACTIN*, 351 bp) and male embryos, by the production of two bands ((*B-ACTIN*, 351 bp) and (*SRY*, 162 bp)). All experiments were completed between July and December 2020.

**Table 1:** Amplification PCR primer sequences of the goat *SRY* and *β-ACTIN* gene

Gene	Accession Number	Primer Sequences (5′→ 3′)	Sizes, bp
*SRY*	NW_017189563	F: TGAACGAAGACGAAAGGTGGCT R: CCTGGGTATTTGTCTCGGTGT	162
*β-ACTIN*	NC_030832	F: CTGTCCCTGTACGCCTCTG R: GCGGAACCGCTCATT	351

### Determination of X/Y sperm ratio by flow cytometry

The proportions of X- and Y-sperm were determined by flow cytometry, as described by Welch et al. (1999) [26]. Briefly, after incubating the sperm with 1 μmol/L R848 for 30 min at 37 °C, the upper or lower sperm layers were centrifuged at 400 *g* at 37 °C for 5 min. The sperm samples were then diluted to a concentration of 2 × 10^8^ sperm/mL with 500 μL of goat extender containing 8 μL of Hoechst 33342 (2 mg/mL) and tailed off by ultrasound for 1 s. The samples were incubated at 37 °C for 50 min in the dark, with mixing every 10 min. A high-speed sorting flow cytometer (BD FACSAria™ III Cell Sorter; BD, Franklin Lakes, NJ, USA) was used to analyse the proportions of X- and Y-sperm. The experiments were completed between July and November 2020.

### Western blot analyses

As described in our previous study [27], to obtain total protein lysates, the sperm were lysed on ice for 30 min using a cell lysis buffer containing 250 μL of RIPA and 2.5 μL of PMSF. A BCA assay kit was used to determine total protein concentrations and the protein samples with loading buffer were boiled at 100 °C for 10 min. Protein samples (20 μg of protein in each lane) were electrophoresed on 10% SDS-polyacrylamide gels. The proteins were separated by PAGE and transferred onto polyvinylidene fluoride (PVDF) membranes. The membranes were then incubated with the indicated primary antibodies. Next, the blots were incubated with the primary antibodies overnight at 4 °C. The primary antibodies used were: phospho-AKT (10176–2-AP; Proteintech Group, Inc., China), phospho-NF-κB (3031 s; Cell Signalling Technology, Boston, MA, USA), phospho-GSK3α/β (9331 s; Cell Signalling Technology), total AKT (ab81283; Abcam), total NF-κB (8242 s; Cell Signalling Technology), and total GSK3α/β (5676 s; Cell Signalling Technology). Finally, after washing with TBST, the blots were incubated with an alkaline phosphatase-conjugated secondary antibody (1:10,000 dilutions in TBST) for 1 h at 37 °C. The reactive proteins were visualised using chemiluminescence (ECL) western blot reagents and quantified using ImageJ software. The levels of the target proteins were normalised to those of anti-alpha tubulin (ab7291; Abcam), which was used as an internal control. The experiments were completed between July and November 2020.

### Statistical analyses

All results were expressed as the mean ± standard error of the mean (SEM). Before statistical analysis, all data were tested for normal distribution and homogeneity of variance. Percentage data were transformed by arc-sin square root transformation to normalise the distributions prior to statistical analysis. Statistical analyses of data from at least three replicates were compared using either Student’s t-test or one-way ANOVA, and multiple comparisons were performed with Duncan’s multiple range test using SPSS version 20 for Windows (SPSS Inc., Chicago, IL, USA) (*, *P* < 0.05; **, *P* < 0.01).

## Results

### Localisation of TLR7 and TLR8 in dairy goat testes, epididymis, and sperm

**Fig. 2:**
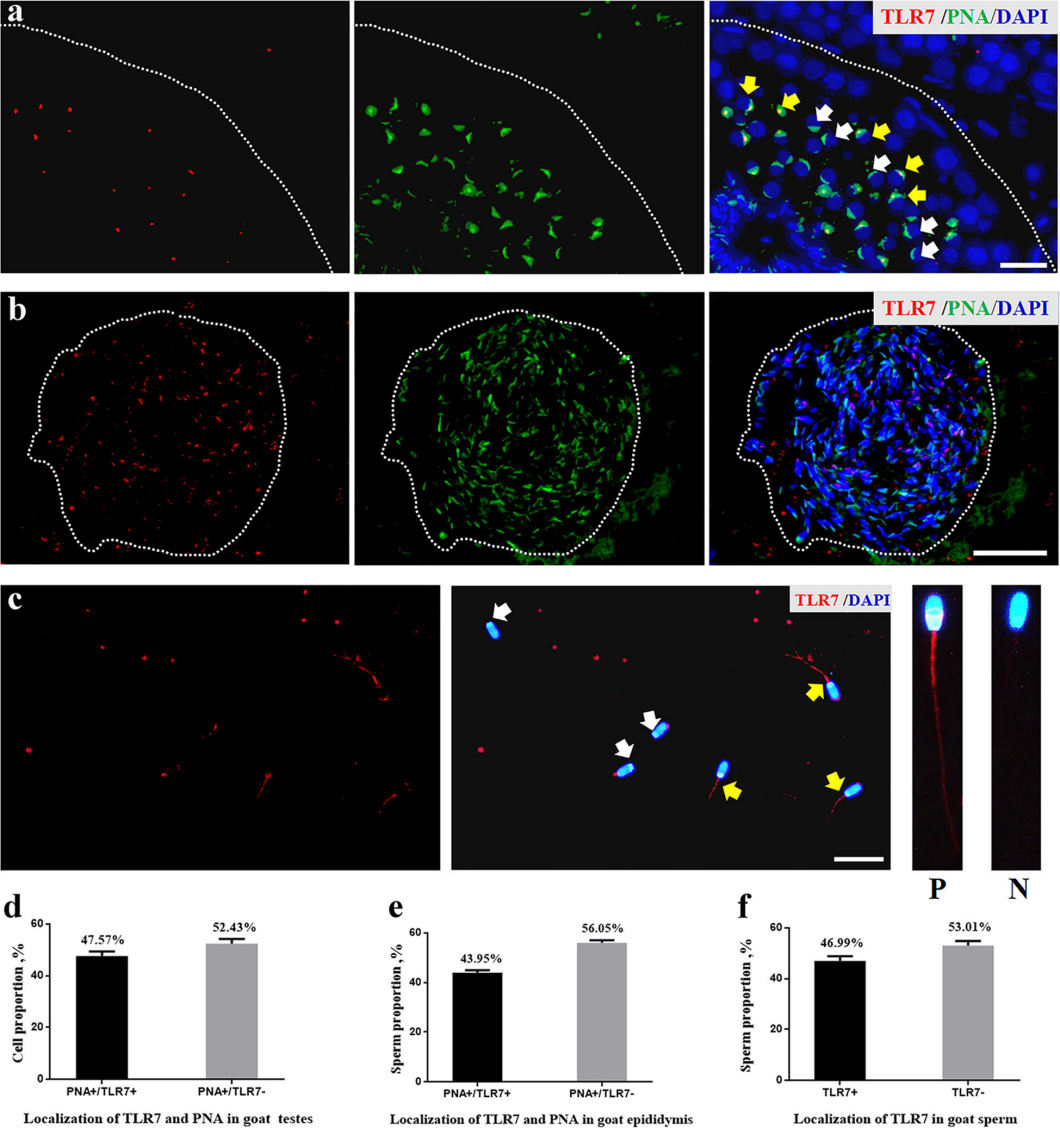
The percentage and localisation of TLR7 in goat testes, epididymis and sperm. a, localisation of TLR7 in goat testes at 8 months age. Cross-sections of goat testes were stained with TLR7 (red) or PNA (green), a marker of acrosome in round spermatids and sperm. Yellow arrows represent PAN^+^ and TLR7^+^ positive cells, white arrows represent PAN^+^ and TLR7^−^ positive cells. Scale bar indicates 20 μm. b, localisation of TLR7 in goat epididymis at 8 months age. Cross-sections of epididymis were stained with TLR7 (red) or PNA (green). Scale bar indicates 50 μm. c, localisation of TLR7 in goat sperm collected by ejaculation, TLR7^+^ signals (red) were observed at the entire tail of the sperm. Scale bar indicates 20 μm. Yellow arrows represent TLR7 positive sperm (P), white arrows represen TLR7 negative sperm (N). d, the percentage of TLR7 positive/negative cell in goat testis. e, the percentage of TLR7 positive/negative sperm in goat epididymis. f, the percentage of TLR7 positive/negative sperm in goat sperm. Bars represent the mean ± SEM (*n* = 3)

**Fig. 3:**
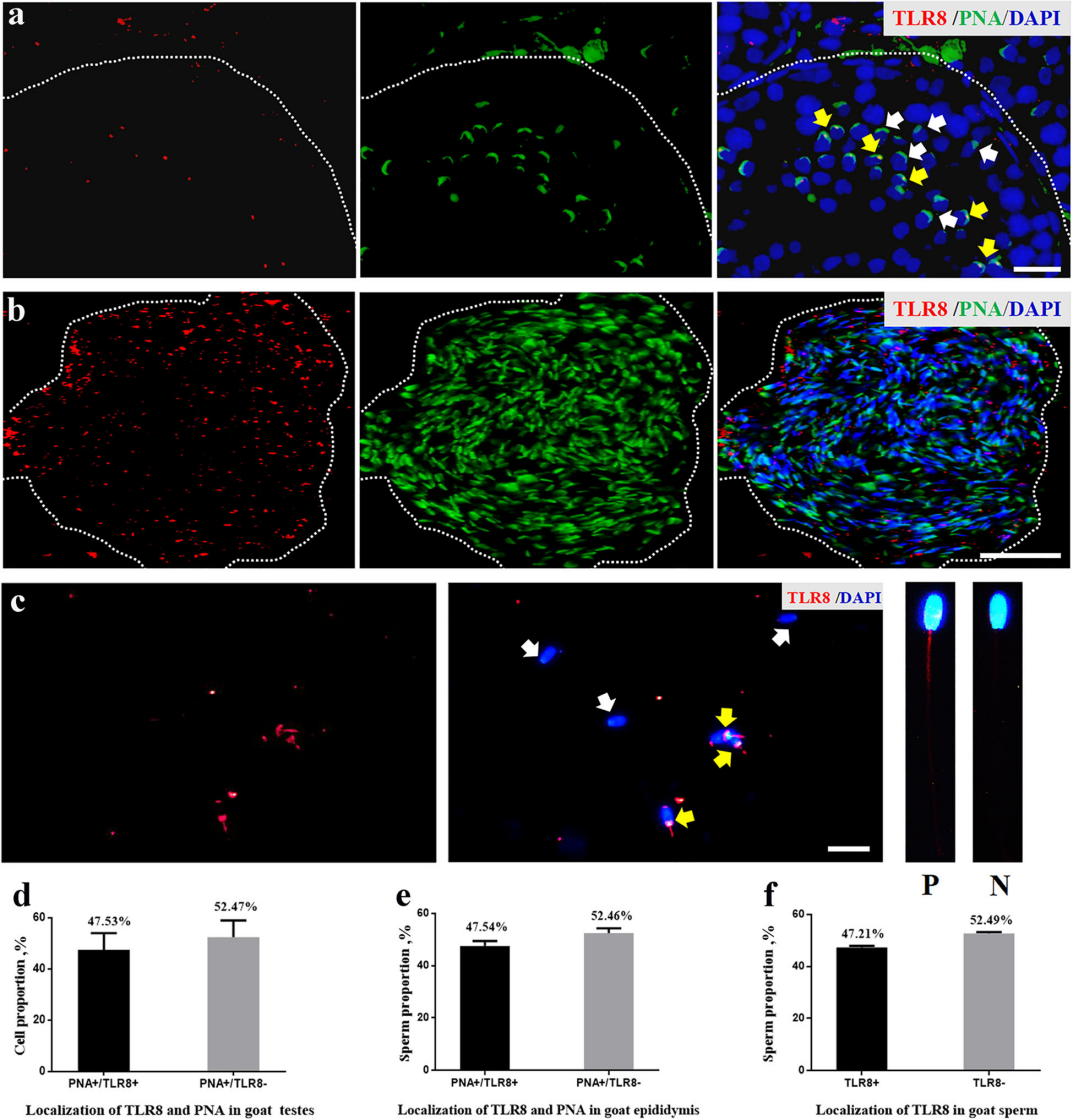
The percentage and localisation of TLR8 in goat testes, epididymis and sperm. a, localisation of TLR8 in goat testes at 8 months age. Cross-sections of goat testes were stained with TLR8 (red) or PNA (green), a marker of acrosome in round spermatids and sperm. Yellow arrows represent PAN^+^ and TLR8^+^ positive cells, white arrows represent PAN^+^ and TLR8^−^ positive cells. Scale bar indicates 20 μm. b, localisation of TLR8 in goat epididymis at 8 months age. Cross-sections of epididymis were stained with TLR8 (red) or PNA (green). Scale bar indicates 50 μm. c, localisation of TLR8 in goat sperm collected by ejaculation, TLR8^+^ signals (red) were mainly localised at the connecting piece and midpiece of the sperm tail. Scale bar indicates 20 μm. Yellow arrows represent TLR8 positive sperm (P), white arrows represent TLR8 negative sperm (N). d, the percentage of TLR8 positive/negative cell in goat testis. e, the percentage of TLR8 positive/negative sperm in goat epididymis. f, the percentage of TLR8 positive/negative sperm in goat sperm. Bars represent the mean ± SEM (n = 3)

The percentage and localisation of TLR7 and TLR8 in dairy goat testes, epididymis, and sperm are shown in Figs. 2 and 3. Using immunofluorescence techniques, TLR7^+^ cells were observed in approximately half of the round spermatids (47.57 ± 1.79%) and epididymis sperm (43.95 ± 1.02%) stained with PNA, a special dye that specifically binds to the acrosome (Fig. 2a, b, d, and e). TLR7^+^ signals (red) were observed at the entire tail of the sperm in 46.99 ± 1.83% of the total goat buck sperm collected after ejaculation (Fig. 2c and f). On the other hand, TLR8^+^ signals (red) comprised 47.53 ± 6.54% and 47.54 ± 1.87% of PNA^+^ round spermatids and epididymis sperm, respectively (Fig. 3a, b, d, and e). In addition, the results showed that the TLR8^+^ signals (red) were mainly localised at the connecting piece and midpiece of the sperm tail (Fig. 3c). Consistently, the proportion of TLR8^+^ sperm collected after ejaculation was approximately 50% of the total sperm (Fig. 3c and f).

### TLR7/8 agonists reduced the values of the goat sperm motility parameters

**Fig. 4:**
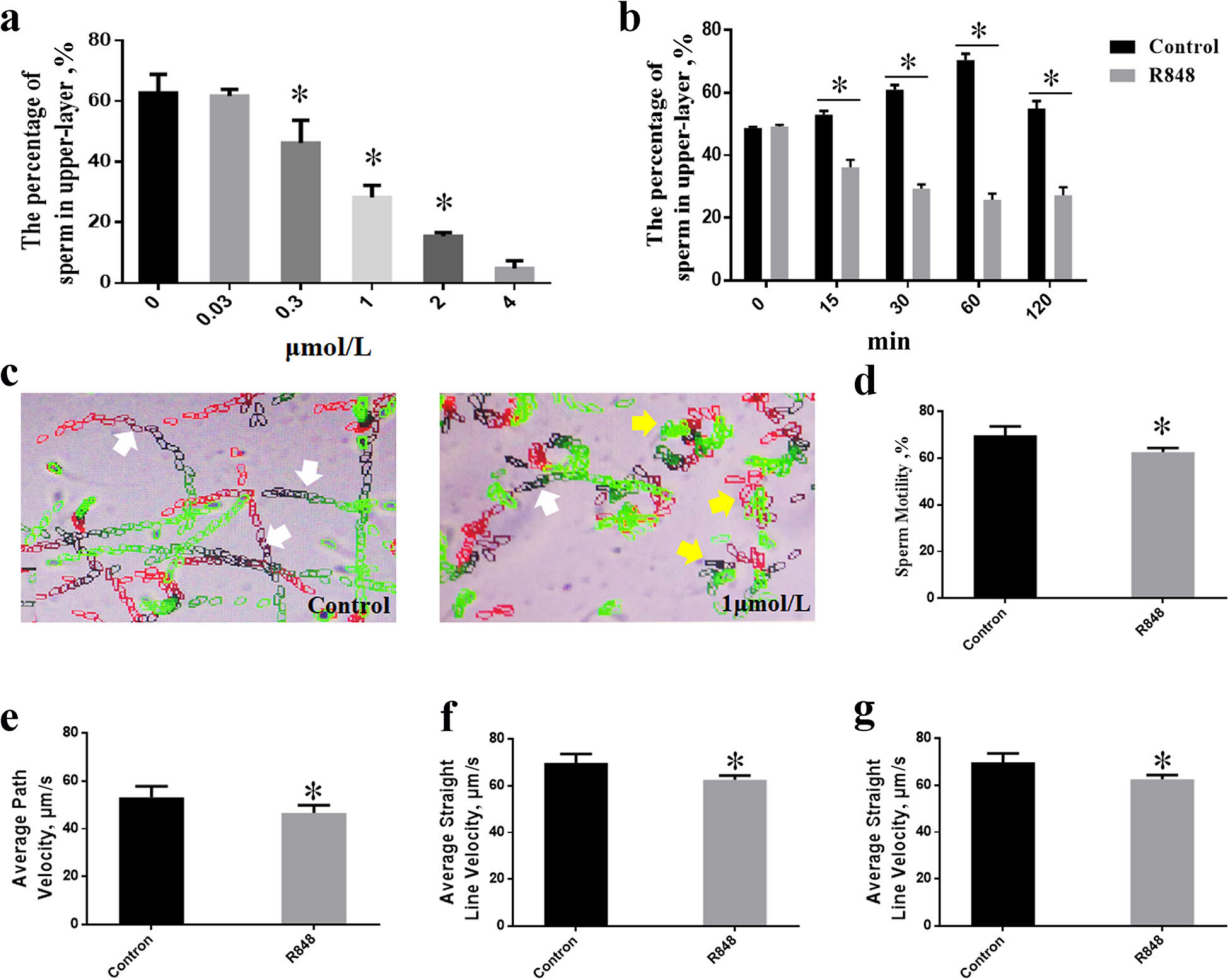
TLR7/8 agonists inhibit goat sperm motility parameters. a, the percentages of swim-up sperm when sperm were cultured in extender with R848 for 60 min at 37 °C. The numbers of sperm in the upper layer and the number of total sperm were counted at 1 h. b, the percentages of swim-up sperm when sperm were cultured in extender with 1 μmol/L R848. Sperm were cultured in extender with 1 μmol/L R848 for a maximum of 120 min. The numbers of sperm in the upper layer and total sperm were counted at each time point. The percent of swim-up sperm was then calculated. c, Tracks of sperm incubated with 1 μM R848 for 30 min, determined using the CASA system. The white arrow indicated the sperm moving forward (progressive sperm). The yellow arrow indicated the sperm swinging in a circle (slow sperm). d, e, f and g, sperm were incubated with 1 μmol/L R848 for 30 min. Sperm motility, average path velocity (VAP), average straight line velocity (VSL), and average curve line velocity (VCL) were measured using CASA system, respectively. Bars represent the mean ± SEM (*n* = 5). *P < 0.05 compared with the control

The effects of TLR7/8 agonists (R848) on sperm motility parameters were evaluated, and these results are shown in Fig. 4. As shown in Fig. 4a, after the sperm (3 mL, 1 × 10^8^ sperm/mL) were incubated with different concentrations of R848 for 60 min at 37 °C, the percentage of highly active goat sperm that swam to the upper layer (1 mL) was significantly reduced by the addition of R848 in a dose-dependent manner (from 0.3 to 4 μmol/L), and was significantly lower than that in case of the control group. In particular, sperm motility was also significantly reduced compared to the control group after incubation with 1 μmol/L R848 for 60 min (Fig. S1). Additionally, compared with the control group, the percentage of sperm in the upper layer of semen (1 mL) decreased after incubation with 1 μmol/L R848 for more than 15 min, in a time-dependent manner (Fig. 4b). However, there was no significant reduction in sperm motility compared to the control group when the semen was incubated for 15 min with 1 μmol/L R848 (S1 Fig). Sperm motility parameters were analysed using the CASA system, and interestingly, the trajectories of goat sperm incubated with 1 μmol/L R848 for 30 min were shorter than those of the sperm from the control group (Fig. 4c). Meanwhile, after incubation for 30 min, 1 μmol/L R848 significantly decreased the values of the sperm motility parameters, i.e., the VAP, VSL, and VCL, of the goat sperm, compared with the corresponding values of the sperm in the control group (*P* < 0.05) (Fig. 4d–g).

### TLR7/8 agonists suppressed the motility of the sperm bearing the X-chromosome

**Fig. 5:**
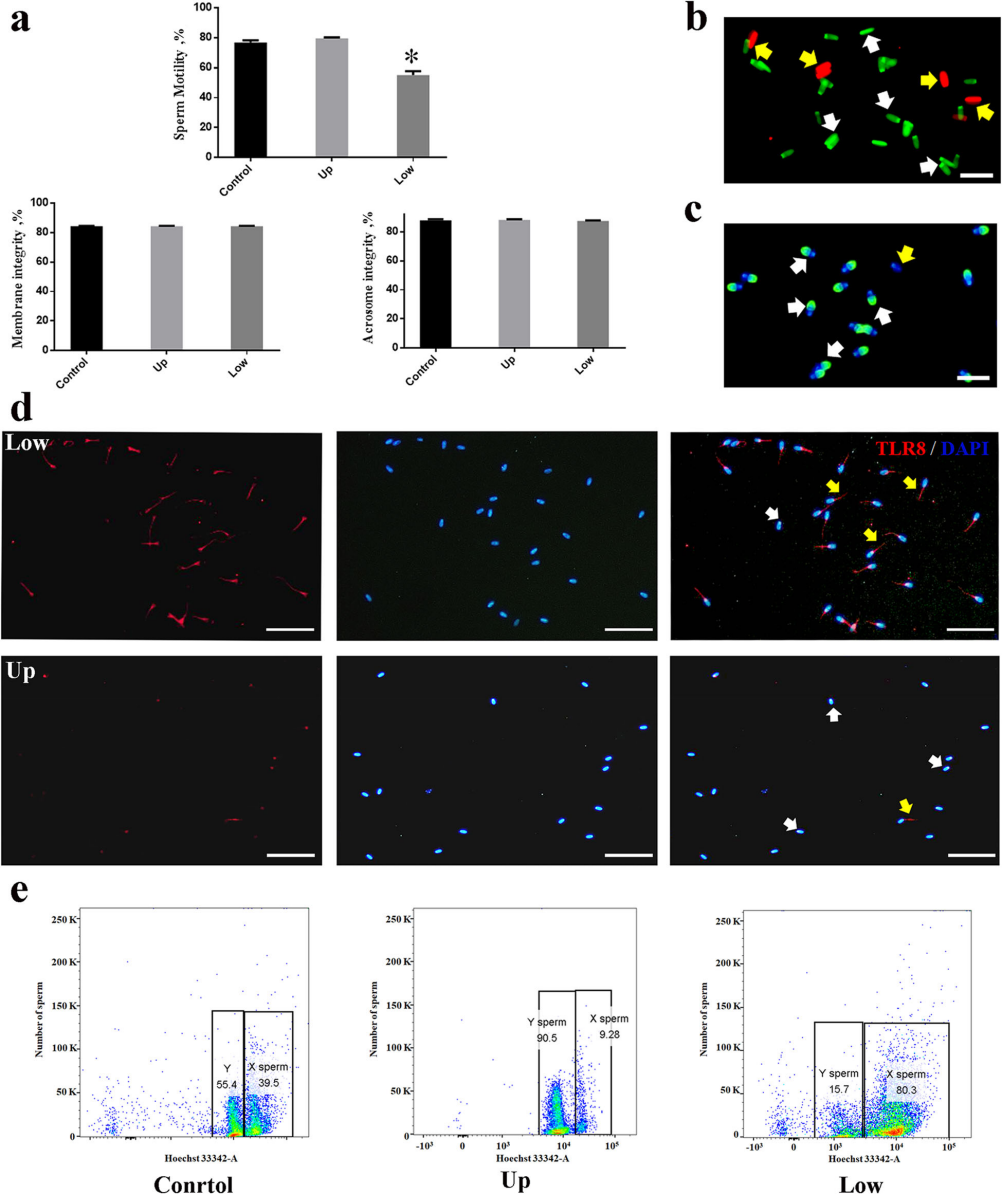
The percentage of Y-sperm and X-sperm in the upper layer and lower layer sperm after TLR7/8 agonists treatment. a, comparison of sperm motility, membrane integrity, and acrosome integrity in the upper layer and lower layer sperm with 1 μmol/L R848 for 30 min (n = 5). b, sperm were stained using SYBR-14/PI. White triangle indicates membrane integrity; yellow arrows indicates membrane damaged (Bars = 20 μm, n = 5). c, sperm were stained using FITC-PNA. White triangle indicates acrosome integrity; yellow arrows indicates acrosome damaged (Bars = 20 μm, n = 5). d, localisation of TLR8 in the upper layer and lower layer goat sperm after incubate with 1 μmol/L R848 for 30 min. Yellow arrows represent TLR8 positive sperm, white arrows represen TLR8 negative sperm (Bars = 50 μm). e, the percentage of X-sperm and Y-sperm in the upper layer and lower layer sperm were measured using flow cytometer system, values are the mean ± SEM of three replicates. *P < 0.05 compared with the control

The motility of the sperm in the upper and lower layers after incubation with 1 μmol/L R848 for 30 min was evaluated. After the swim-up test, compared with the upper-layer sperm and control sperm groups, the motility of sperm in the lower layer was dramatically decreased (Fig. 5a). Interestingly, the membrane and acrosome integrity of the upper- and lower-layer sperm were not reduced compared to the sperm from the control group (Fig. 5a). After washing to remove R848, there was no significant reduction in the motility of the lower-layer sperm, compared to the case for the sperm from the control group (Fig. S2). Observations using IF showed that the lower-layer sperm had more TLR8^+^ signals than the upper-layer sperm (Fig. 5d). The genes *TLR7* (Gene ID 102171227) and *TLR8* (Gene ID 100860909), from the National Coalition Building Institute, were encoded on the X-chromosome of dairy goats and were expressed in the sperm. The percentages of Y- and X-sperm in the upper- and lower-layer sperm were assessed using flow cytometry, based on the DNA contents. The percentage of the Y-sperm in the upper layer was 90.50% ± 2.86% and that of the X-sperm in the lower layer was 80.30% ± 2.91% (Fig. 5e).

### TLR7/8 agonists reduced the ATP levels and mitochondrial activity via NF-κB and GSK3α/β phosphorylation

**Fig. 6:**
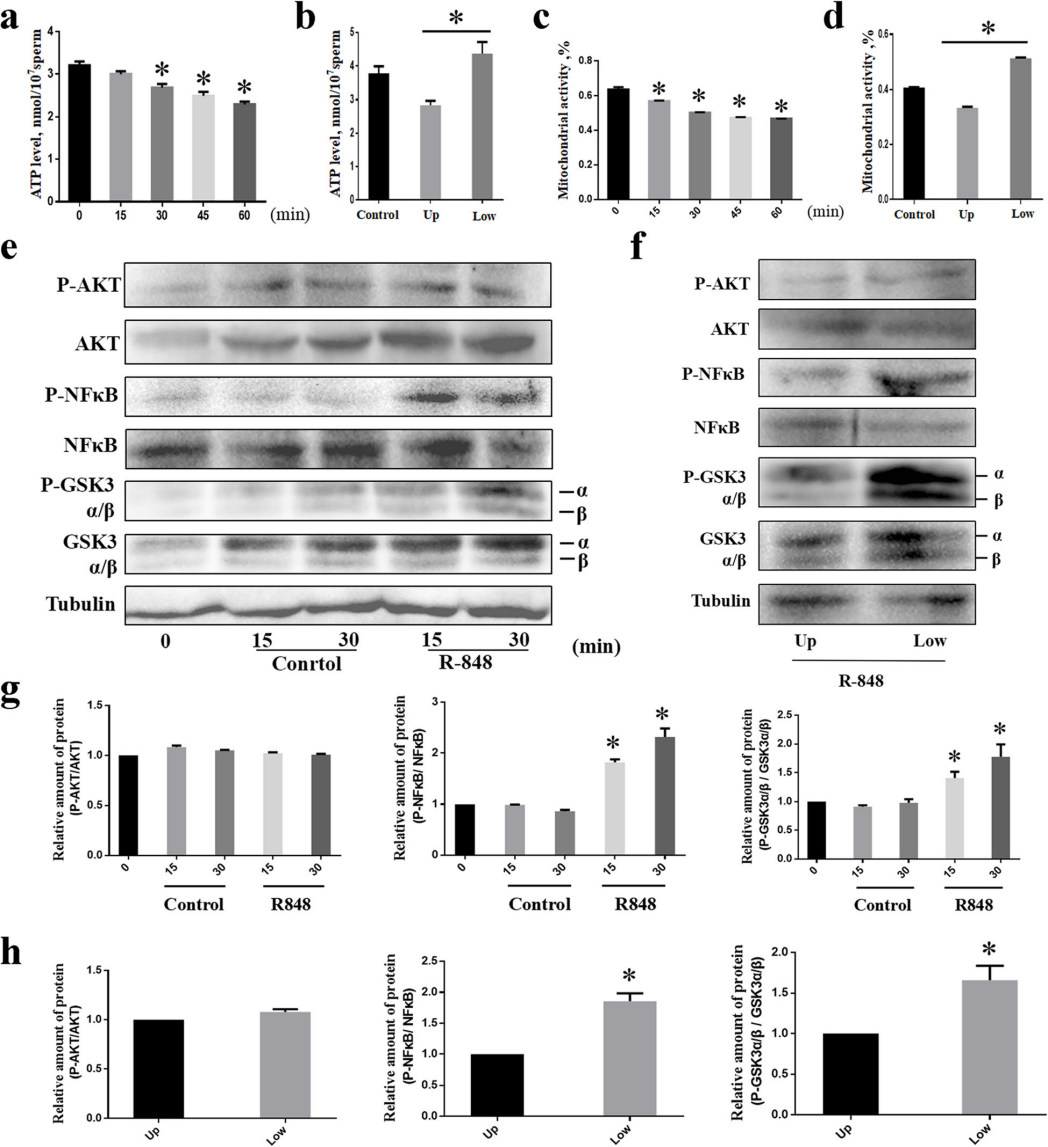
TLR7/8 agonists inhibited ATP production via GSK3 α/β-hexokinase pathway. a, the ATP levels of sperm after culture in extender with 1 μmol/L R848 for a maximum of 60 min. b, the ATP levels of sperm in the upper layer and lower layer sperm with 1 μmol/L R848 for 30 min. c, the mitochondrial activity of sperm after culture in extender with 1 μmol/L R848 for a maximum of 60 min. d, the mitochondrial activity of sperm in the upper layer and lower layer sperm with 1 μmol/L R848 for 30 min. e, goat sperm were incubated with 1 μmol/L R848 for 0, 15, or 30 min, and 20 μg of protein lysates was analyzed for phospho-AKT, phospho-NFκB, phospho-GSK3α/β, total AKT, total NFκB, and total GSK3α/β expression by Western blotting. Tubulin was used as the loading control. Results are representative of three independent experiments. f, intensity of the phosphorylation of AKT, NFκB, and GSK3α/β in the upper layer and lower layer sperm induced by R848 for 30 min. g and h, columns represent the ratio of phosphorylated to total AKT, phosphorylated to total NFκB or phosphorylated to total GSK3α/β. Values are the mean ± SEM of three replicates. *P < 0.05 compared with the control

Mitochondria are important sources of energy for sperm motility. Thus, we evaluated the effects of the TLR7/8 ligand on ATP production and mitochondrial activity in sperm. As shown in Fig. 6a, the ATP levels of sperm decreased after incubation with 1 μmol/L R848, in a time-dependent manner, for more than 30 min. Mitochondrial activity was decreased after incubation with 1 μmol/L R848 for over 15 min, compared to case for the sperm in the control group (Fig. 6c). After the swim-up test, the ATP levels in the lower-layer sperm were significantly reduced compared to those of the upper-layer sperm (Fig. 6b). The mitochondrial activity of the lower-layer sperm was consistently significantly lower than that of the upper-layer sperm (Fig. 6d). Using western blot analysis, increased levels of NF-κB and GSK3α/β phosphorylation were detected in the sperm treated with R848 for 15 and 30 min (Fig. 6e and g), compared with the sperms from the control group. However, there was no effect of this treatment on AKT phosphorylation (Fig. 6e and g). These changes were observed in the lower-layer sperm (X-sperm) after incubation with 1 μmol/L R848 for 30 min, as per the results of the swim-up test (Fig. 6f and h). These data confirm that TLR7/8 agonists affect X-sperm motility via NF-κB and GSK3α/β phosphorylation-mediated regulation of ATP levels and mitochondrial activity.

### TLR7/8 ligand activation enabled sex control for IVF and AI

**Fig. 7:**
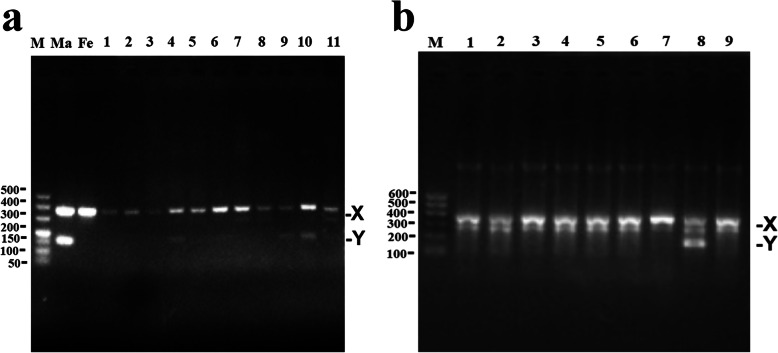
TLR7/8 agonists inhibited X-sperm motility and enabled sex control for in vitro fertilization (IVF) and artificial insemination (AI). a, gel of duplex PCR products of DNA extracted from single embryo after IVF, using *SRY* and *B-ACTIN* primers, respectively. Lane M is 500 bp DNA ladders; lane Ma is the male goat DNA; lane Fe is the female goat DNA; lanes 1, 2, 3, 5, 6, 7, 8 and 11 are female embryos; lanes 4, 9, and 10 are male embryos. b, gel of duplex PCR products of DNA extracted from single embryo after AI, using *SRY* and *B-ACTIN* primers, respectively. Lane M is 600 bp DNA ladders; lanes 1, 2, 3, 5, 6,7 and 9 are female embryos; lane 8 is male embryo

The operation method for separating dairy goat X- and Y-sperm is shown in Fig. 1. After the swim-up test, the X-sperm (lower layer) separated by R848 treatment were collected and used for IVF and AI. Forty-three embryos were obtained by IVF using the lower-layer sperm. Thirty-five of these were XX embryos (80.52% ± 6.75%) and eight were XY embryos (19.48 ± 6.75%) (Fig. 7a). Using the lower-layer sperm, six dairy goat does were simultaneously subjected to superovulation and oestrus, and five were artificially inseminated. Nine embryos were collected from the uteri of the two does that conceived. Eight of these were XX embryos and one was an XY embryo (88.89% XX embryos; Fig. 7b). Since only nine embryos were obtained by artificial insemination, these results can only be considered as preliminary findings.

## Discussion

Mammalian sperm are male germ cells that carry genetic information, fertilise oocytes, and determine the sex of the offspring. According to Mendelian separation, the ratio of X- to Y-sperm produced by meiosis in mammalian spermatogenesis is 1:1 [6]. Therefore, the proportions of male and female offspring produced by natural mating are also expected to be equal. Since the economic benefits of animal husbandry are often associated with livestock sex, the ability of farmers to predetermine livestock sex could increase profitability [1]. Hence, sexing control of offspring in livestock is crucial for scientific research and production practice. Thus, this study aimed to demonstrate a simple and novel dairy goat sperm sexing technology that activates TLR7/8 signalling-induced inhibition of X-sperm motility. Our results showed, for the first time, that TLR7/8 was expressed in sperm carrying the X-chromosome in dairy goats, and that treating X-sperm with TLR7/8 ligands (R848) could reduce the motility of X-sperm, without affecting the Y-sperm. These results indicate that X- and Y-sperm from dairy goat bucks can be separated by targeting X-sperm expressing TLR7/8.

TLRs, which are members of an evolutionarily ancient pattern-recognition receptor family, play a key role in mammalian innate immunity and its association with inflammation [28]. A previous report indicated that TLR signalling affects sperm motility and mitochondrial function in mice [29]. In the present study, TLR7 and TLR8 were investigated to clarify their potential function in the sperm of dairy goat bucks. We found that TLR7/8 was located in the sperm tail, and the proportions of TLR7^+^ and TLR8^+^ sperm in the testes, epididymes, and ejaculate of dairy goats were nearly equal. Umehara et al. (2019) also demonstrated that the percentages of TLR7^+^ and TLR8^+^ sperm were approximately 50% each in the cauda epididymis of mice, which is consistent with the results of the present study [13]. Furthermore, both *TLR7* (Gene ID 102171227) and *TLR8* (Gene ID 100860909) are specifically expressed on the X-chromosome in dairy goats. Therefore, we hypothesised that TLR7/8 may be expressed explicitly in the X-sperm.

R848 (resiquimod), a small-molecule compound of imidazoquinoline, is an agonist of TLR7 and TLR8 that is currently used clinically to treat infectious viral diseases and tumours [30]. In the present study, we demonstrated that R848 reduced sperm motility, VAP, VSL, and VCL. These are crucial kinematic characteristics of sperm: significant correlations have been identified between VAP and the number of acrosome-reacted spermatozoa [31]; VAP and VSL bear a positive correlation with fertility [32]; and VCL is relevant to sperm concentration and affects fertility [33]. Therefore, R848 may impair sperm movement by interfering with the TLR7/8 pathway in vitro. However, after removing R848, sperm motility was restored, indicating that the inhibition of sperm motility by R848 is reversible, which agrees with the findings of Umehara et al. (2019) [13]. In the present study, after incubating goat sperm with R848, the sperm were separated into the upper and lower layers for further analysis. Interestingly, the motility of the lower-layer sperm was significantly reduced while that of the upper-layer sperm remained unchanged. After labelling the sperm with Hoechst 33342, reanalysis of the sorted samples by flow cytometry could be used to distinguish X/Y sperm with high precision [26]. O’Brien et al. (2005) used flow cytometry to reanalyse the proportion of the X- and Y-sperm of four primate species and considered that this is a reliable method for detecting the sperm ratio with low additional costs [34]. Goat sperm in the upper and lower layers were evaluated by flow cytometry, and the proportion of Y- or X-sperm reached more than 80%, respectively. These results further support our hypothesis that TLR7/8 is expressed exclusively in X-sperm.

These findings indicate that R848 inhibits X-sperm motility by specifically activating the TLR7/8 pathway, which mediates motility-associated functional differences between X- and Y-sperm in dairy goat bucks. As reported in our previous study, membrane and acrosome integrity were significant parameters for evaluating sperm function and quality [17]. In the present study, the membrane and acrosome integrity of sperm in the upper or lower layers was not significantly reduced after incubation with 1 μmol/L R848 for 30 min. Therefore, we speculated that the reduction of X-sperm motility by R848 may be related to the mitochondrial function and ATP levels. Tourmente et al. (2019) reported that ATP is essential for sperm motility in mice and showed that increasing ATP contents were linked with faster sperm that were more efficient swimmers [35]. The mitochondrial volume in goats has recently reported to be positively correlated with sperm swimming velocities and ATP levels [36]. In our study, as the time duration of the incubation with 1 μmol/L R848 increased, the mitochondrial activity and ATP levels in the goat sperm were reduced. Notably, only the mitochondrial activity and ATP levels of X-sperm (lower-layer sperm) were significantly decreased by 1 μmol/L R848. Our results corroborate those of a study by Umehara et al. [13]. Therefore, we provide the first evidence that TLR7/8 signalling affects X-sperm motility via the direct regulation of ATP levels in dairy goat bucks. However, further studies are required to elucidate the mechanisms whereby TLR7/8 regulates ATP levels in goat sperm.

**Fig. 8:**
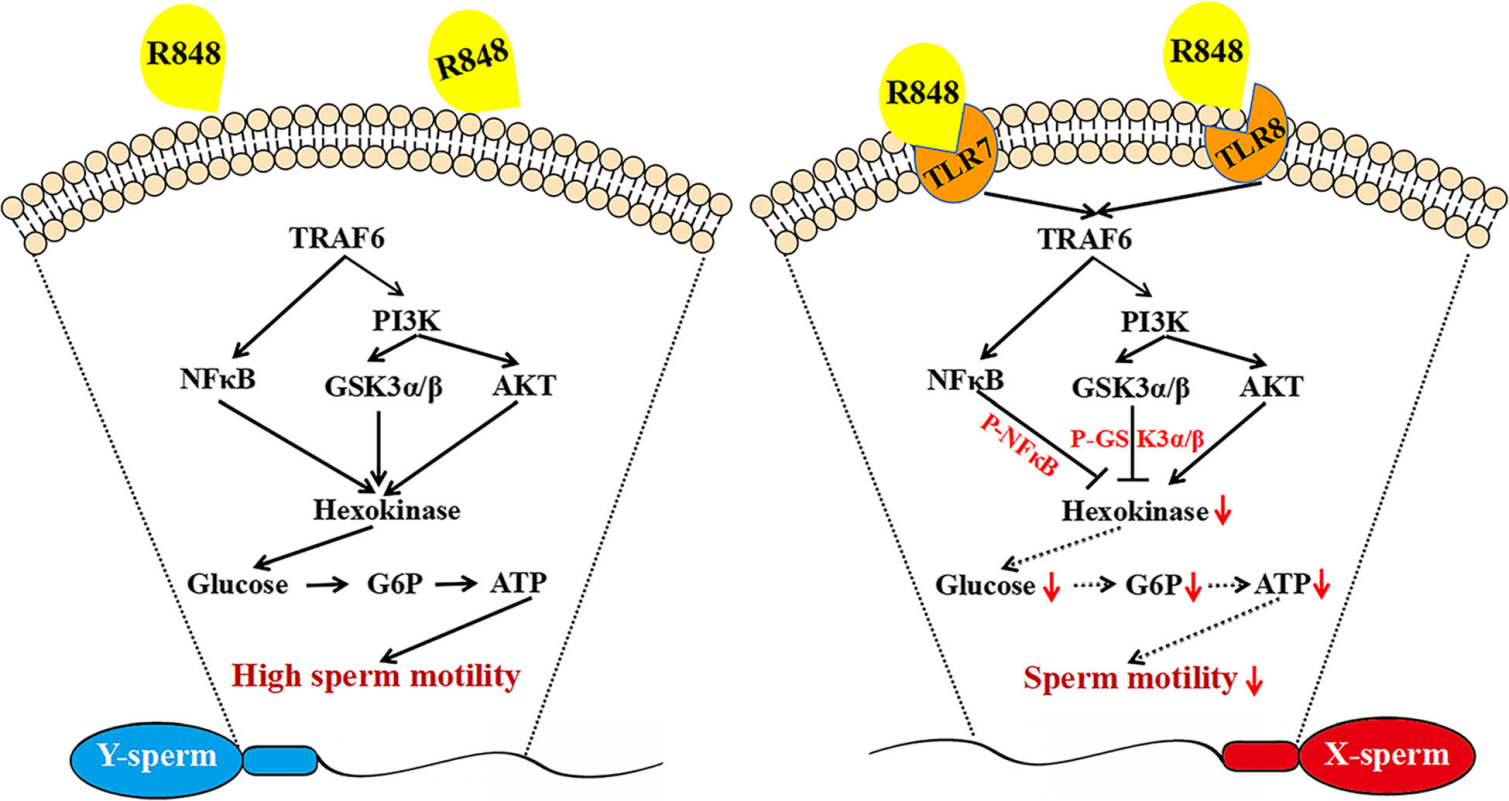
TLR7/8 signalling mechanisms affect ATP production in X-sperm and Y-sperm of goat, respectively. In Y-sperm, mitochondrial and glycolytic ATP production occur regardless of the presence of R848. Thus, Y-sperm showed normal high-speed motility. In X-sperm, the activation of TLR7/8 localised in the sperm tail via phosphorylated NFκB and GSK3α/β, and via a PI3K/AKT-independent mechanism. Then suppressed the activity of hexokinase. As a result, ATP production in X-sperm was decreased under R848 condition. Thus, only X-sperm showed low-speed motility under R848 condition

The maintenance of sperm motility depends on complex interactions between different cell signalling systems, one of which is a network of signalling pathways involved in regulating sperm movement [37]. The phosphoinositide 3-kinases (PI3Ks), a family of lipid kinases, participate in multiple signalling pathways in cells, including cell proliferation, migration, survival, metabolism, and intracellular trafficking [38]. AKT is a key downstream stimulant of PI3K and is closely related to sperm motility [39, 40]. Li et al. (2020) focused on the relationship between sperm motility and PI3K/AKT activity in mice and found that sperm motility decreased notably after blocking AKT activity [41]. In the current study, the goat sperm incubated with R848 showed decreased motility, while AKT phosphorylation was unaffected. This observation implies the possible involvement of other signalling pathways in R848 metabolism. Sperm motility in mice was shown to decrease after TLR signal activation, but as observed in our study, AKT was not phosphorylated [29]. Further, a previous study in mice indicated that TLR7/8 signalling affects sperm motility and is associated with reduced ATP levels [13] due to the phosphorylation of glycogen synthase kinase 3α/β (GSK3α/β) and the activation of nuclear factor-kappa B (NF-κB), which are well-known downstream effectors of TLR7/8 [13]. We report this phenomenon for the first time in goats. Based on the results of the western blotting analysis, we hypothesised that TLR7/8 signal activation could improve the protein expression levels of phosphorylated GSK3α/β and NF-κB through the TRAF6/PI3K/GSK3α/β and TRAF6/NF-κB pathways, respectively. Moreover, TLR7/8 signalling decreased the ATP levels via the suppression of hexokinase activity in goat sperm (Fig. 8). However, further studies are needed to verify this hypothesis.

Based on these observations, R848 was used to reduce the ATP levels of X-sperm and inhibit X-sperm motility, while Y-sperm motility was unaffected. This phenomenon could be used to separate X- and Y-sperm in dairy goats. We referred to the sperm separation method in mice as reported in the study by Umehara et al. (2019) and achieved similar results in dairy goats [13]. The ratio of X-sperm to Y-sperm, after sorting using this method, reached more than 80% in dairy goats, and the motility of the X-sperm could be restored after the R848 treatment was stopped. In fact, scientists have been trying to find a way to differentiate between and separate X- and Y-sperm [6]. At present, flow cytometry is the most widely used method for separating sperm in livestock, with a separation accuracy of over 90% [42]. However, due to the differences in the sperm of different species of animals, this method has only been commercially applied to separate X/Y sperm in cattle [7]. In other domestic species, including goats and pigs, this method has only been tested on a small scale [10, 43]. In the present study, the percentage of female embryos obtained by in vitro fertilisation with separated X-sperm was 80.52 ± 6.75%, suggesting that the sex-sorted sperm could fertilise oocytes, and the resulting XX embryos had a similar ratio to that of sorted X-sperm. More encouragingly, the sorted X-sperm could produce more XX embryos after AI in the cervix. Since the AI experiment was conducted during the non-breeding season (winter) of goats and only nine embryos were obtained, it is difficult to draw strong conclusions regarding the true in vivo efficacy of the X-sperm after sorting. However, this was within an acceptable range, considering that 88.89% of XX embryos were obtained after the AI process. Bathgate et al. (2013) indicated, for the first time, that goat sperm could be sex-sorted by flow cytometry, successfully producing five female kids (83%) [44], which is consistent with our findings and indicates that the sperm sorted using our proposed method could pass through the cervix to fertilise oocytes in vivo. Thus, the ratio of X- to Y-goat sperm after sorting by this method was over 80%, and the motility of the X-sperm was restored after the inhibitor was removed. In addition, incubating sperm with TLR7/8 ligands permitted the easy separation of X- and Y-sperm, resulting in more than 80% of the embryos being female when produced using X-sperm during IVF. These data indicate that TLR7/8 signalling sufficiently affects X-sperm motility to separate X- and Y-sperm and can be therefore, be used to efficiently produce sexed dairy goat embryos.

## Conclusions

In conclusion, a novel, simple sperm-sexing method for dairy goats that activates TLR7/8-inhibited X-sperm motility to separate X- and Y-sperm was investigated. Our study showed that TLR7/8 signalling affected X-sperm motility via the GSK3 α/β-hexokinase pathway, which is important for the efficient production of sexed dairy goat embryos.

## Supplementary Information

**Additional file 1: S1 Figure.** TLR7/8 agonists inhibit goat sperm motility. a, the sperm motility was measured using CASA system after cultured in extender with different concentrations R848 for 1 h at 37 °C. b, the sperm motility was measured using CASA system after cultured in extender 1 μmol/L R848 for a maximum of 120 min at 37 °C. Bars represent the mean ± SEM (*n* = 5). *P < 0.05 compared with the control.

**Additional file 2: S2 Figure.** The recovery of sperm motility suppressed with R848 by centrifugation. Sperm (3 mL, 1 × 10^8^ sperm/mL) were incubated with 1 μmol/L R848 for 30 min, and then lower-layer sperm (1 mL) were collected to a new tube. After centrifuging, the sperm pellet was washed R848-free extender, the sperm motility before/after washing was compared by CASA. Bars represent the mean ± SEM (n = 5). *P < 0.05 compared with the control.

## Data Availability

All data generated or analyzed during this study are available from the corresponding author by request.

## Abbreviations

TLR7/8: Toll-like receptor 7/8
GSK3 α/β: Glycogen synthase kinase α/β
NFκB: Nuclear factor kappa-B
AI: Artificial insemination
X-sperm: X Chromosome bearing sperm
Y-sperm: Y Chromosome bearing sperm
PI: Propidium iodide
IVF: In vitro fertilization
CASA: Computer-aided sperm analysis
IF: Immunofluorescence
PNA-FITC: Fluorescein isothiocyanate-peanut agglutinin
PCR: Polymerase chain reaction
TLRs: Toll-like receptors
NCBI: National Coalition Building Institute
