# Supplementary figures and images for: TLR7/8 signalling affects X-sperm motility via the GSK3 α/β-hexokinase pathway for the efficient production of sexed dairy goat embryos
Source: J Anim Sci Biotechnol. 2021 Aug 3;12:89. doi: 10.1186/s40104-021-00613-y (PMC8330071; doi:10.1186/s40104-021-00613-y)

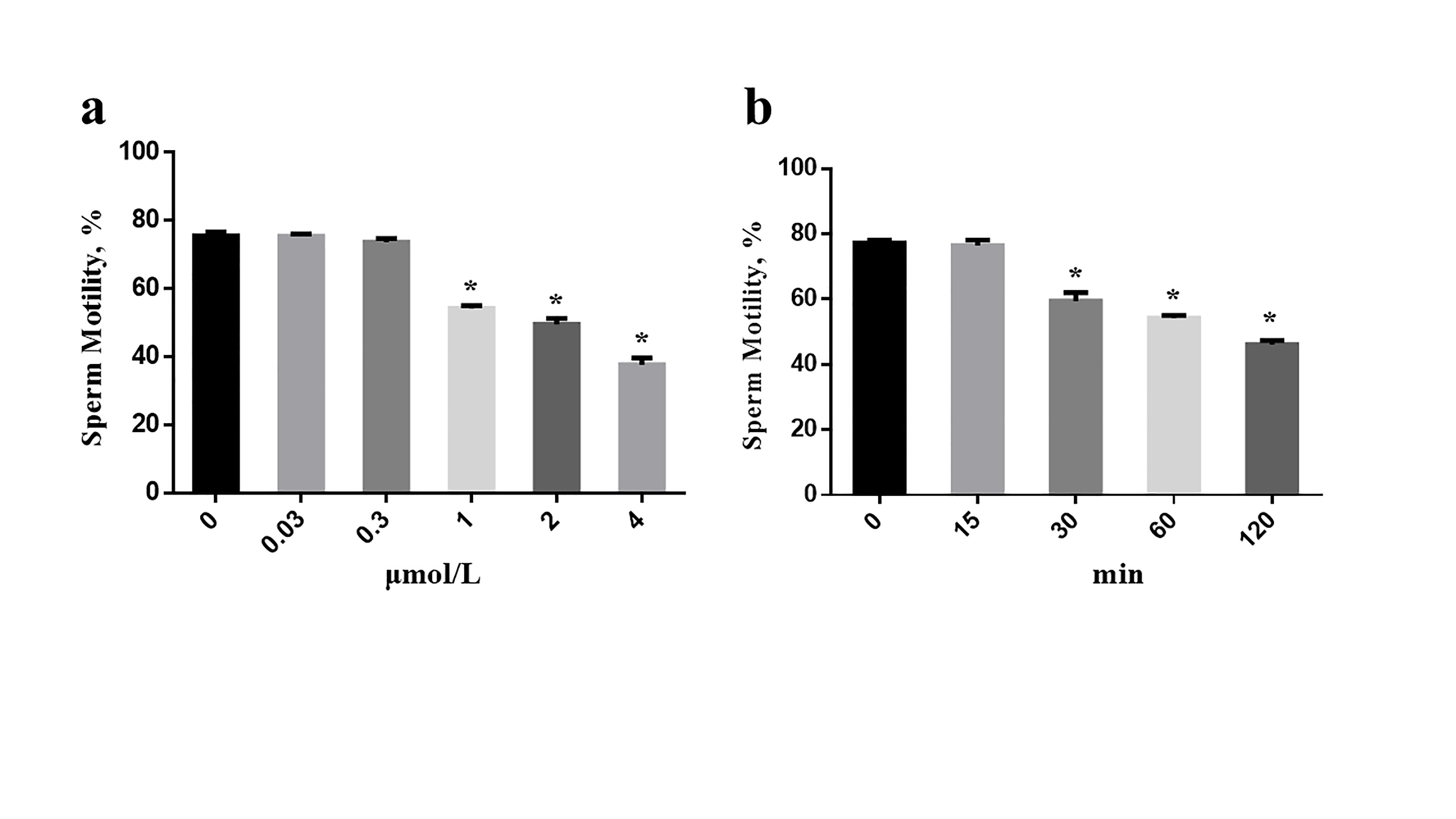

Supplement: Supplementary file 1 — Additional file 1: S1 Figure. TLR7/8 agonists inhibit goat sperm motility. a, the sperm motility was measured using CASA system after cultured in extender with different concentrations R848 for 1 h at 37 °C. b, the sperm motility was measured using CASA system after cultured in extender 1 μmol/L R848 for a maximum of 120 min at 37 °C. Bars represent the mean ± SEM (n = 5). *P < 0.05 compared with the control. [file 40104_2021_613_MOESM1_ESM.tif]

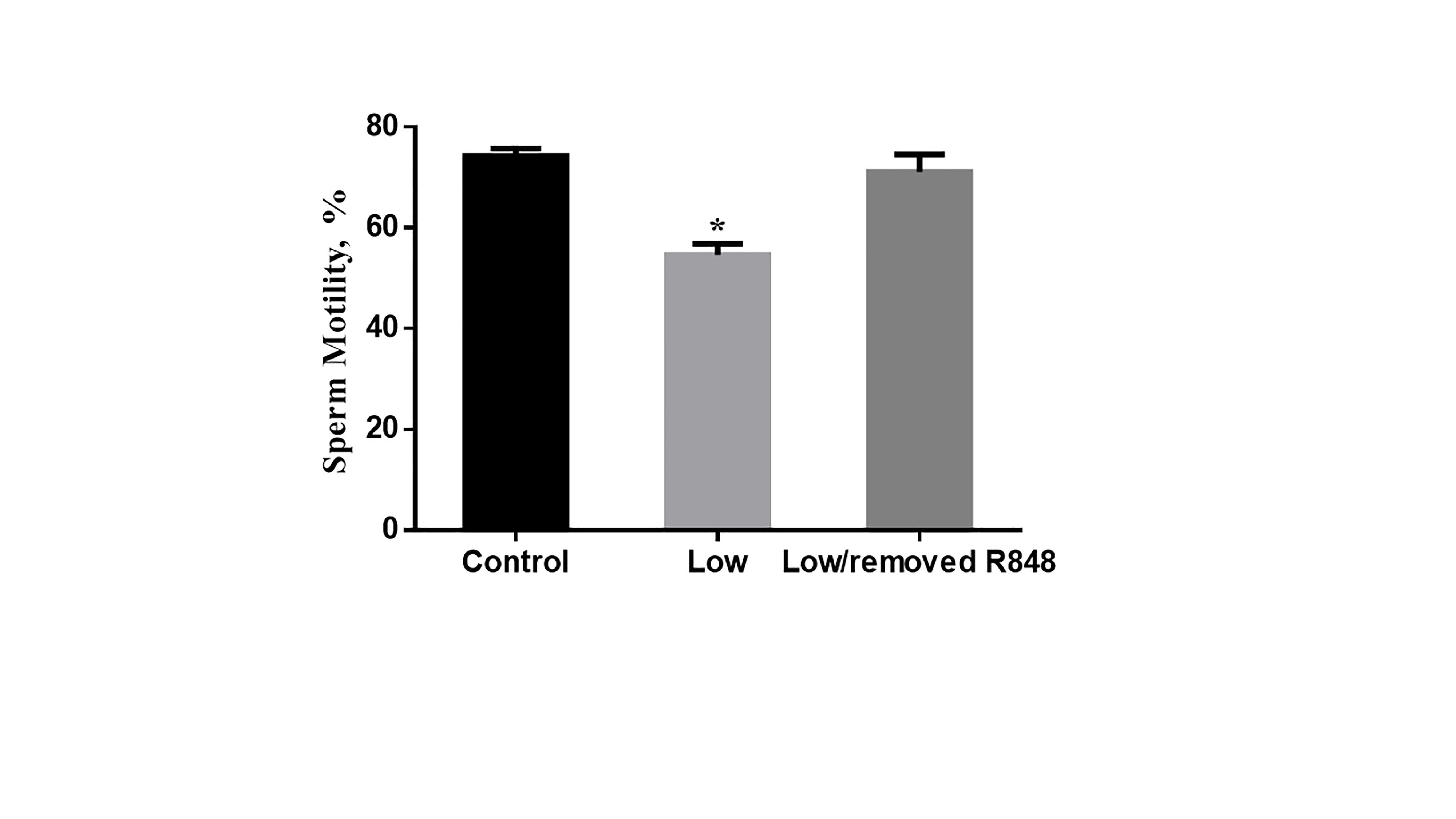

Supplement: Supplementary file 2 — Additional file 2: S2 Figure. The recovery of sperm motility suppressed with R848 by centrifugation. Sperm (3 mL, 1 × 10^8^ sperm/mL) were incubated with 1 μmol/L R848 for 30 min, and then lower-layer sperm (1 mL) were collected to a new tube. After centrifuging, the sperm pellet was washed R848-free extender, the sperm motility before/after washing was compared by CASA. Bars represent the mean ± SEM (n = 5). *P < 0.05 compared with the control. [file 40104_2021_613_MOESM2_ESM.tif]
